# Returns to scale in food preparation and the Deaton–Paxson puzzle

**DOI:** 10.1007/s11150-017-9399-4

**Published:** 2017-12-02

**Authors:** Thomas F. Crossley, Yuqian Lu

**Affiliations:** 1 Department of Economics, University of Essex, Wivenhoe Park, Colchester, C04 3SQ UK; 2 Institute for Fiscal Studies, Bloomsbury, London UK; 3 Statistics Canada, 100 Tunney’s Pasture Driveway, Ottawa, ON K1A 0T6 Canada

**Keywords:** Household returns to scale, Home production, Food preparation, Time use, D11, D12, D13

## Abstract

We consider returns to scale in food preparation as a potential resolution of a puzzle raised by Deaton and Paxson (Journal of Political Economy, *106*(5), 897–930, 1998). We clarify the conditions under which returns to scale in food preparation can resolve the puzzle. The key requirement is that foods are heterogeneous in time costs. We then show that detailed food expenditure and time use data are consistent with larger households shifting to more time intensive foods.

## Introduction

In an influential paper, Deaton and Paxson (1998) raise an important puzzle in understanding returns to scale in household consumption. They note that, holding per capita resources constant, returns to scale (in at least some goods) imply that larger households are better off, and so should consume more of private goods such as food. However, they document in a range of data sets that larger households have *lower* per capita food expenditures (holding per capita resources constant).

Gan and Vernon (2003) suggest that returns to scale in food consumption—particularly in food preparation—may resolve this puzzle. However, Deaton and Paxson (1998, 2003) emphasize that the presence of returns to scale in food preparation strengthens rather than resolves their puzzle.^1^

The latter assertion is true of models in which “food” is a single commodity, or at least homogeneous with respect to preparation time. In contrast, if food is a composite of goods that differ in their preparation times, returns to scale in food preparation are a potential explanation of the Deaton–Paxson Puzzle. The first contribution of this article is to develop a slightly richer model that retains the Barten-type demographic effects of Deaton and Paxson’s analysis but adds explicit home production (of food) and two types of food (which differ in their preparation times). Thus it serves to illustrate the case in which returns to scale in food preparation might explain the Deaton– Paxson Puzzle, and contrasts with the case in which food is homogeneous with respect to time costs. Deaton and Paxson recognize this possibility in their original paper, but do not consider it a likely explanation (Deaton and Paxson 1998, p. 922).

The second contribution of this article is to compare some of the predictions of a model with foods that are heterogeneous in preparation times to Canadian data from detailed food expenditure diaries and detailed time use diaries. We find the data are consistent with key predictions of our model. Compared to singles, couples spend a smaller share of their food budget on prepared food, and, strikingly, couples spend more time per capita on food preparation than singles.

With respect to the Deaton–Paxson puzzle, we find that a version of the puzzle remains. However, we argue that the type of test for returns to scale that Deaton and Paxson propose may be difficult to implement (at least for developed countries) because of substitutions *within* broad food categories.

In the next section we set out the background to our analysis by reviewing the puzzle posed by Deaton and Paxson (1998), and the implications of adding home production to their model while maintaining the assumption that food is a homogeneous good. In Section 3, we contrast this analysis with a model with two types of food that differ in their preparation times. Section 4 presents our empirical evidence. Section 5 discusses alternative explanations for the patterns we see in the data, and implications of our findings.

## Background

### The Deaton–Paxson puzzle

Consider a household with *n* members (adults only) who enjoy two goods: a private good *f* (food) and a composite of other goods *x* which is subject to some scale economies. To focus on returns to scale, we follow Deaton and Paxson (1998) in assuming a unitary model of household preferences. The household’s problem is:

$$\max \,nu\left( {\frac{f}{n},\frac{x}{{n^\theta }}} \right)\quad 0 < \theta < 1$$

$$st.\quad p_{f}\frac{f}{n} + p_x\frac{x}{n} = \frac{y}{n}$$

The expression *n* ^*θ*^ captures returns to scale in the (composite) good *x*. When $n = 1$, $n^\theta = 1^\theta = 1$; when $n = 2$, $n^\theta = 2^\theta < 2$, and so on.^2^ ^3^ The *p*_*i*_ are market prices and *y* is household income (or total expenditure).

Note that the budget constraint can be rewritten in terms of individual consumption, per capita income, and *shadow* prices:

$${p_{f}^{\ast}} \; f{\ast} + {p_{x}^{\ast}} \; x{\ast} = y{\ast},$$

where

$$y\ast = \frac{y}{n},\;f\ast = \frac{f}{n},\; {x {\ast}} = \frac{x}{{n^\theta }},$$

and

$${p_{f}^{\ast}} = p_f,\;\quad {p_{x}^{\ast}} = \frac{{p_x}}{{n^{1 - \theta }}}.$$

The key insight of Barten-type models (Barten 1964) is that demographics have price-like effects. Here, as household size increases, the price of the private good (food) is unaffected.^4^ In contrast, the resource cost (shadow price) of the good subject to returns to scale (x*) falls: $\frac{{\partial {p_{x}^{\ast}}}}{{\partial n}} < 0$. This will have both income and substitution effects. For the public good, these operate in the same direction (the household is richer, and the relative price of public goods is lower). Thus as *n* increases, x* (individual consumption of *x*) should unambiguously rise. However, note that x* is not observed by the econometrician, as it depends on the returns to scale parameter *θ*. In contrast, individual food consumption, *f* *, is observed (as it depends only on total food and household size.) The income and substitution effects for food have opposite sign (the household is better off, but the relative price of food has risen), but Deaton and Paxson (1998) posit that since there are few substitutes for food, the income effect should dominate.^5^ Thus, holding resources (income or total outlay) per capita constant, larger households should have higher per capita consumption of food and, given common market prices, higher per capita food expenditures.

Deaton and Paxson examine expenditure data from a range of countries and find the opposite result: larger households have lower per capita food expenditures holding per capita income constant. They consider and reject a number of possible explanations for this puzzle.

### Food preparation with homogeneous time costs

Beside food, Deaton and Paxson (1998) also examine household expenditures on some other private goods. They find that “the coefficients on household size are generally positive for clothing and entertainment”,^6^ which implies that food differs from other private goods in some crucial way. Gan and Vernon (2003) suggest that returns to scale in food consumption would help resolve the puzzle and speculate that returns to scale in the time cost of food preparation might be the source of returns to scale in food consumption.

Deaton and Paxson (2003) respond that (direct) returns to scale in food consumption could certainly help to explain the puzzle but note that returns to scale in the time required for food preparation actually deepen, rather than resolve, the puzzle. To see why, consider the following simple extension to the model, adding home production (of food):

$$\max \,nu\left( {\frac{f}{n},\frac{x}{{n^\theta }},\frac{l}{n}} \right)$$

$$\begin{array}{ccccc}\\ s.t.: \frac{f}{n} = \min \left[ {\frac{t}{{n^\gamma }},\frac{i}{n}} \right]\\ \\ w\left( {\frac{T}{n} - \frac{l}{n} - \frac{t}{n}} \right) = p_x\frac{x}{n} + p_i\frac{i}{n}\\ \\ 0 < \theta < 1,\quad 0 < \gamma < 1\\ \end{array}$$

where *i* is quantity of ingredients purchased, *w* is wage rate, *T* is the total time endowment, *t* is time spent on food preparation, *l* is leisure time, and *T* is total time endowment. Optimization implies:

$$t = \frac{i}{{n^{1 - \gamma }}}$$

and:

$$f = i$$

so that the problem can be rewritten:

$$\max \,nu\left( {\frac{i}{n},\frac{x}{{n^\theta }},\frac{l}{n}} \right)$$

$$s.t.:\quad w\frac{T}{n} = p_x\frac{x}{n} + p_i\frac{i}{n} + w\frac{l}{n} + w\frac{i}{n}\left( {\frac{1}{{n^{1 - \gamma }}}} \right).$$

As before the budget constraint rewritten in terms of individual consumption, per capita income (now *full income*), and shadow prices:

$$wT\ast = {p_{x}^{\ast}} \ x \ast + wl \ast + p_{_{i}}^\ast \ i \ast,$$

where

$$i\ast = \frac{i}{n},\quad l\ast = \frac{l}{n},\quad x\ast = \frac{x}{{n^\theta }},$$

and

$${p_{i}^{\ast}} = p_i + \frac{w}{{n^{1 - \gamma }}},\quad {p_{x}^{\ast}} = \frac{{p_x}}{{n^{1 - \theta }}}.$$

Now the shadow prices (full costs) of both food and other goods (x*) fall with household size, leading to larger income effects. Moreover, the direction of substitution effects, if any, depend on the relative size of the returns to scale parameters *θ* and *γ*, and could favour food. As household size increases (holding resources per capita constant), per capita food consumption—and hence per capita quantities of ingredients purchased—should increase. As Deaton and Paxson note, budget data record *market* expenditures on ingredients (food). That is, they record $p_ii$, not the full cost, $p_i^\ast i$. However, this is actually useful because (assuming common market prices) market expenditures are proportional to quantities, so per capita market expenditures should rise with household size (holding per capita resources constant). Thus the Deaton–Paxson puzzle remains—and is deepened because income effects should be greater here than in the simpler model.

## Food preparation with heterogeneous time costs

Models in which foods differ in their time cost have quite different implications. The simplest model that illustrates this point assumes that there are just two kinds of food, with the most extreme heterogeneity in time costs of preparation. Prepared or “cooked” food, *c*, is purchased “ready-to-eat” and requires no preparation time. Alternatively, ingredients *i* can be purchased and combined with time to produce regular food, *r*. We assume the same home production technology is as in the model of the previous section. We do not assume that prepared food and home cooking are perfect substitutes. Thus in this model the household’s problem is:

$$\max \,nu\left( {\frac{f}{n},\frac{x}{{n^\theta }},\frac{l}{n}} \right)$$

$$\begin{array}{ccccc}\\ s.t.:\quad & \frac{{w(T - l - t)}}{n} = p_c\frac{c}{n} + p_i\frac{i}{n} + p_x\frac{x}{n}\\ \\ & \frac{f}{n} = f\left( {\frac{r}{n},\frac{c}{n}} \right)\\ \\ & \frac{r}{n} = \min \left[ {\frac{t}{{n^\gamma }},\frac{i}{n}} \right]\\ \end{array}$$

The production function implies $t = \frac{i}{{n^{1 - \gamma }}}$ and $r = i$. Thus the problem can be written:

$$\begin{array}{l}\max \,nu(f(c \ast,i \ast),x \ast,l \ast)\\ s.t.:wT \ast = {p_{x}^{\ast}} \ x \ast + wl \ast + p_{_i}^\ast i \ast + p_c c\ast\end{array}$$

where

$$c\ast = \frac{c}{n},\quad i \ast = \frac{i}{n},\quad l \ast = \frac{l}{n},\quad x \ast = \frac{x}{{n^\theta }},$$

and

$${p_{i}^{\ast}} = p_i + \frac{w}{{n^{1 - \gamma }}},\quad {p_{x}^{\ast}} = \frac{{p_x}}{{n^{1 - \theta }}}.$$

When household size increases, the shadow prices of ingredients (regular food), $i\ast( = r \ast)$, and other goods, x*, fall. We follow Deaton and Paxson in assuming that substitution effects between food and other goods are negligible (so the change in ${p_{x}^{\ast}}$ affects food purchases only through income effects). The income and substitution effects on food purchased can be summarized as follows. For ingredients, the income and substitution effect operate in the same direction. A larger household is better off, and faces a lower relative (shadow) price of home cooked food. For prepared foods, the income and substitution effects operate in opposite directions, so that the total effect is ambiguous.

There are three key predictions. First, as household size increases there should be a substitution from ready-to-eat or prepared foods towards ingredients. This is important because it means that, across household size, (per capita) market expenditures on all foods are not proportional to (per capita) food quantities. Market expenditures (per capita) are:

$$p_c\frac{c}{n} + p_i\frac{i}{n}$$

If $p_i < p_c$ (as seems reasonable) then substitution from *c* to *i* could lead market expenditures to fall, even if per capita quantities of food were constant or rising. Thus this kind of compositional effect could explain the Deaton–Paxson puzzle. Another way to think about this point is that in this model, the “market price” of food (which in this model is a weighted average) is not constant across household sizes, because households of different size purchase different food baskets. Thus broad expenditure patterns across household sizes are not necessarily informative about quantity patterns.

The second key prediction is that in this model, per capita quantities of the most time intensive food should rise with household size (holding per capita resources constant). This is because of both income and substitution effects. This prediction is in some sense the analogue of the prediction that Deaton and Paxson examine in their original (1998) paper.

Finally, we can use the production condition $\left( {t = \frac{i}{{n^{1 - \gamma }}}} \right)$ to eliminate ingredients rather than preparation time. This leads to an unambiguous prediction that $t \ast = \frac{t}{{n^\gamma }}$ should rise with household size. Of course, t* is not observed in the data because (except for singles) it depends on the return of scale parameter γ. Only *t* (or $\frac{t}{n}$) is observed. However, note that if returns to scale are operating $(0 < \gamma < 1)$ and per capita time ($\frac{t}{n}$) rises with household size, then *effective* time per capita ($t \ast = \frac{t}{{n^\gamma }}$) must rise with household size because $\frac{t}{{n^\gamma }}  >\frac{t}{n}$ when $n \ge 2$. Thus the observation that *per capita* time spent on food preparation rises with household size would support the model—and suggest significant substitution of home cooking for prepared foods in response to differences in shadow prices.^7^ We now turn to an empirical examination of the predictions of our model.

## Some empirical evidence

We next compare predictions of the model described in the previous section to two cross-sectional data sources. The first is the 1992 and 1996 Canadian Food Expenditure Survey (FOODEX), a detailed two-week diary of household food expenditures, which distinguishes several hundred types of food purchased from stores. We have divided those types of foods into "ingredients" (foods requiring substantial preparation) and prepared or “ready-to-eat” foods.^8^ The second data set is the detailed time use diaries that are part of the 1998 Canadian General Social Survey (GSS), and in particular the information on time spent on food preparation in that data.

In our empirical analysis we focus on adult-only households: singles and couples (without children). We further restrict the sample to individuals aged 25–55 and working full time (i.e., both members of a couple must satisfy these criteria for the household to be included). Our FOODEX sample contains 1201 singles and 956 couple households. The GSS sample includes 861 singles and 550 couple households.

We focus on fully employed adult households for two reasons. First, as Deaton and Paxson note, children likely have lower food needs than adults, so that food demand may *fall* as the fraction of children in the household rises (holding per capita income and household size constant.) Thus to look for evidence of returns to scale we should vary household size while holding the fraction of children in the household constant. Our sample of adult only households does this. In our Canadian data, there is limited scope to independently vary household size and the fraction of children at larger household sizes.

Second, as explained below, the limitations of our data make it convenient to focus on households with small (market) labour supply elasticities.

A downside of our sample choice is that when they compare households of size 1 and 2 only, Deaton and Paxson do not find their puzzle in all the countries they consider.^9^ However, we will demonstrate below that the Deaton–Paxson puzzle is evident in our sample.

In both data sets there are slightly more men than women among singles. In all our calculations, we use weights to undo this discrepancy (so that there is no difference in “average” gender between the couples and singles.)^10^

We examine expenditure patterns in the FOODEX data with parametric (OLS) regressions. These relate expenditure shares and ratios of expenditures to the logarithm of income per capita and a couple dummy, as well as additional controls for age, gender and education of the household head. We also include year, season and region fixed effects.^11^ Similar results are obtained by running nonparametric regressions of shares on log per capita income separately for singles and couples.^12^

Note that we condition on total market income, whereas in a home production model like that developed in the previous section the correct conditioning variable is *full* income (the sum of time endowments, each multiplied by the relevant wage). Unfortunately neither data set contains information on wages.^13^ The assumption that maps our theory into our empirical work is that the individuals we study (young, childless, singles and couples, working full time) have inelastic supply of market time (so that we can treat market income as essentially exogenous.) We believe that for this sample, this is a reasonable assumption, and this is the second reason we focus on younger, adult-only households.

We begin in Table 1 by examining food shares. Holding per capita income constant, the estimated shares of couples are lower and the difference from singles is statistically significant (the coefficient on the couple dummy in Column 1 of Table 1). Thus the Deaton and Paxson puzzle is apparent in our data.

**Table 1:** Food expenditure regressions

	(1)	(2)	(3)	(4)
	Food (purchased from store) budget share	Ratio of prepared food to ingredients	Ratio of take-out fast-food to ingredients	Ingredients budget share
	Regression Coefficients × 100 [t-statistics in square parentheses]			
Couple dummy	−1.06 [−3.80]	−10.29 [−4.01]	−10.97 [−3.21]	−0.73 [−2.96]
ln(per capita income)	−8.87 [−6.13]	−2.11 [−0.76]	6.55 [1.74]	−7.41 [−5.72]
R^2^	0.31	0.030	0.020	0.28

Based on a pooled sample of 1201 singles and 956 childless couples from the 1992 and 1996 Canadian Food Expenditure Surveys. All members are aged 25–55 and working full time. In all calculations the data are weighted to equalize the proportion of each gender amongst singles

Additional regressions controls include age, sex, education of the household head, as well as year, season and region fixed effects

Full results are presented in the appendix

We now turn to an analysis of prepared foods and ingredients. Holding per capita resources constant, food expenditures of couple households are significantly shifted towards ingredients (and away from prepared foods). The difference between couples and singles is statistically significant (Column 2 of Table 1). These are the substitution patterns within food predicted by our extended model.

In addition to prepared food purchased for consumption at home, we also examine expenditures on take-out fast-food. Take-out fast-food can be considered food at home with little preparation time (perhaps even less than the prepared foods purchased in stores).^14^ Column 3 of Table 1, illustrates that, holding per capita resources constant, food expenditures of couple households are significantly shifted away from fast-food and towards ingredients, which is again consistent with our first prediction.

These types of substitutions suggest the possibility of substantial returns to scale in food preparation, and substantial responses to the resulting differences in shadow prices across household types. They also mean that food at home is not a homogeneous commodity, and that across household sizes, expenditures are not proportional to quantities. If larger households are substituting towards foods that are cheaper on the market but require greater time inputs, then *market* expenditures may fall, while quantities do not fall, or even rise.

Some evidence on this is provided in Tables 2 and 3, which focus particularly on meat purchases. Meat is a useful category of food for this analysis because it is frequently bought in both prepared and ingredient form. The FOODEX data collects both expenditures and quantities, so that we can examine quantities directly. We can also calculate unit values, which are expenditure divided by quantity – similar to a price.^15^

**Table 2:** Unit values, prepared and unprepared meat

	(1)	(2)
	Unit value unprepared meat ($)	Unit value prepared meat ($)
Singles	6.55 (0.12)	7.92 (0.17)
Couples	6.25 (0.11)	8.17 (0.16)

Based on a pooled sample of 959 singles and 884 childless couples with positive meat purchases from the 1992 and 1996 Canadian Food Expenditure Surveys. All members are aged 25–55 and working full time. In all calculations the data are weighted to equalize the proportion of each gender amongst singles

**Table 3:** Meat unit values, expenditures and quantities

	(1)	(2)	(3)	(4)	(5)
	Share of prepared meat in total meat ($)	Share of prepared meat in total meat (kgs)	Unit value total meat ($)	Total meat $ per capita	Total meat Kgs per capita
	Regression coefficients × 100 [t-statistics in square parentheses]				
Couple dummy	−6.32 [−4.08]	−7.22 [−4.65]	−20.68 [−1.56]	−129.43 [−2.69]	−17.78 [−1.71]
ln(per capita income)	0.81 [0.43]	0.88 [0.47]	39.50 [2.20]	177.65 [2.94]	20.16 [1.48]
R^2^	0.03	0.03	0.06	0.03	0.03

Based on a pooled sample of 959 singles and 884 childless couples with positive meat purchases from the 1992 and 1996 Canadian Food Expenditure Surveys. All members are aged 25–55 and working full time. In all calculations the data are weighted to equalize the proportion of each gender amongst singles

Additional regressions controls include age, sex, education of the household head, as well as year, season and region fixed effects

Full results are presented in the appendix

Table 2 indicates that, for both singles and couples, the average unit value ($ per kg) of prepared meat is higher than for unprepared meat (an ingredient)—by about 25%. These differences are both economically and statistically significant, and thus confirm, at least for meat, our assumption (above) that $$p_i < p_c$$.

Table 3 reports a further set of regressions on  the logarithm of per capita income and a couple dummy (with additional controls as in Table 1). The first two columns consider the share prepared meat in total meat purchases either by expenditure (Column 1) or quantity (Column 2). In both columns we show that couples allocate a significantly lower share of their meat spending to prepared meat. Given that prepared meat is more expensive than unprepared meat (as suggested by Table 2), this implies couples pay a lower average price for all meat. This is confirmed in Column 3, though the effect is not statistically significant at conventional levels.

Again, to explain the Deaton–Paxson puzzle returns to scale in the home production of food must generate a lower average market price for larger households, so that quantities can be higher (as predicted by theory) even though their expenditures are lower (as observed in the data).^16^

An alternative way to look at this is to examine total meat expenditures and quantities directly. This is reported on columns 4 and 5 of Table 3. Couples spend significantly less on meat per capita than singles (column 4), even though with returns to scale they are better off. This echoes the original Deaton–Paxson puzzle. However, column 5 reveals that difference in quantities is much smaller (and not statistically significant). This again suggests that part of the explanation for the Deaton–Paxson puzzle could be that quantities are not proportional expenditures across household sizes.

Returning to ingredients and prepared foods more generally, the second key prediction of our model is that per capita quantities of the most time intensive good—in our case ingredients—should rise with household size (holding per capita income constant). This is because of both the income and substitution effects of the change in shadow prices brought about by increasing household size. Assuming that market prices of ingredients are constant across household size, this means that, at a given level of per capita resources, couple households should spend a larger share of their budget on ingredients. This is not what we observe in our data. Table 1 indicates that couples spend a lower share of their budget on ingredients. Thus a version of the Deaton and Paxson puzzle remains.

Of course, this observation can be explained by the same argument that we have applied to total food expenditures. ‘Ingredients’ in turn are a composite good comprising many types of food with different preparation times, and substitutions between them may mean that market expenditures on ingredients are not proportional to quantities. Couples may pay a lower ‘average’ price because of such compositional effects. However, it is difficult to provide affirmative evidence of this hypothesis (largely because it is not clear what further dis-aggregation of food expenditures would be most appropriate).

Our extended model can be solved for the time input rather than ingredients, and then gives the unambiguous prediction that *effective* time per person on food preparation should rise with household size. A potential empirical problem is that *effective* time is not observed (as it depends on the returns to scale parameter). Nevertheless, Table 4 summarizes food preparation times from the 1998 GSS Time Use Survey.^17^ The key point is that couples spend more time *per person* on meal preparation than singles (the mean is almost 6 min higher; this rises to more than 8 min per day when we add regression controls) so that the total household food preparation time of couples is more than double the food preparation time of singles. We find similar results for food preparation (which includes both meal preparation and shopping) and food preparation plus clean up. As noted above, if per capita time rises moving from singles to couples, then *effective* time must also rise. Thus the time use data are also broadly consistent with the predictions of our model. The fact that couples spend more than twice as much (total) time on food preparation as singles suggests very significant substitution responses to the variation in shadow prices across household types.

**Table 4:** Per capita time use regressions

	(1)	(2)	(3)	(4)
	Food preparation and cleanup	Food preparation (meal preparation + shopping)	Meal preparation	Grocery shopping
Mean minutes per day (Standard Error)				
Singles	40.72 (1.44)	35.55 (1.28)	26.44 (1.00)	9.00 (1.00)
Couples	47.65 (2.29)	39.93 (2.00)	31.97 (1.68)	8.00 (1.00)
Regression coefficients [t-statistics in square parentheses]				
Couple dummy	9.54 [2.78]	6.70 [2.19]	8.85 [3.34]	−2.14 [−1.49]
R^2^	0.07	0.06	0.05	0.02

Based on a sample of 861 singles and 550 childless couples from the 1996 Canadian General Social Survey. All members are aged 25–55 and working full time. The data are weighted to equalize the proportion of each gender amongst singles

Additional regressions controls include age, sex, education of the household head, income category dummies as well as season and region fixed effects

Note that the dependent variable in Column (2) is sum of the dependent variables in Columns (3) and (4). The dependent variable in Column (1) is the sum of the dependent variable in Column (2) and post-meal clean up

Full results are presented in the appendix

## Discussion

In this article we have considered returns to scale in home production of food as a possible explanation of the Deaton–Paxson puzzle. As Deaton and Paxson have suggested, this requires that foods are heterogeneous with respect to time costs. To illustrate this idea we developed develop a slightly richer model that retains the Barten-type demographic effects of Deaton and Paxson’s analysis but adds explicit home production (of food) and two types of food (which differ in their preparation times). Per capita food quantities can be higher in larger households even though their per capita expenditures are lower (as observed in the data) if larger households pay a lower effective market price per unit of food. This would be the case if returns to scale in the time input to home production induced larger households to substitute towards foods that have lower market prices but are more time intensive.

We then compared predictions from this model to detailed Canadian data on food expenditures and on time use. The data align with the predictions of the model in a number of respects. First, using detailed food expenditure data we show that larger households’ food baskets are significantly shifted away from prepared and ready-to-eat foods and towards foods requiring preparation time (‘ingredients’). Second, we provide evidence from time use data that *per capita* food preparation time is significantly greater for working couples than for working singles.

On the other hand, in our extended model with two kinds of foods, market expenditures on the more time intensive type of food should increase with household size, holding per capita resources constant. However, in our data, market expenditures on ‘ingredients’ do not increase with household size (holding per capita resources constant). Deaton and Paxson (1998) noted that *if* food expenditure rose with household size as theory suggests, this would provide a Robarth-type measure of the returns to scale in the household. But as food expenditure does *not* rise with household size (holding per capita income constant) they could not draw an inference about the size of returns to scale in the household. We are in a similar position. Of course, the failure of ingredient expenditures to rise with household size could be explained by the same argument we applied to total food expenditures: ‘Ingredients’ in turn are a composite good comprising many types of food with different preparation times, and substitutions between them mean that market expenditures on ingredients are not proportional to quantities. Larger families may pay a lower ‘average’ price because of such compositional effects. If such substitution patterns are important at finer levels of dis-aggregation, Deaton and Paxson’s strategy for testing for returns to scale may be very difficult to implement.

Further caveats to our analysis are as follows. First, there are other mechanisms that could generate lower effective market prices for larger households (and so explain the Deaton–Paxson puzzle). A greater scope for larger households to take advantage of bulk discounts would do so, but, as noted in the Introduction, the best empirical evidence does not support this explanation for the Deaton–Paxson puzzle. Less food waste in larger households would also mean they face a lower effective market price for food, but we have no direct evidence for or against this possibility in our Canadian data. As Deaton and Paxson (1998) note, this is unlikely to be an explanation for the puzzle in poorer countries.

Second, the kinds of substitution patterns we have considered (between prepared foods and ingredients, or more generally, between foods with different preparation times) may well be much less important in developing countries, or among those living at subsistence levels. Evidence from developing countries was an important part of Deaton and Paxson’s original empirical analysis, and so the mechanism we study is unlikely to be a complete explanation for the puzzle they raise.

Finally, there are other mechanisms that could generate the patterns we see in our data. In particular, it could be that preferences differ between singles and coupled individuals, either because of selection (those who enjoy home cooking partner), or because preferences are contingent on circumstances (it’s more fun to cook when you have someone to cook for.) Our model and interpretation of the data have assumed that preferences are independent of couple status and it is only choice sets that vary with household size. This is a common assumption in the literature (see for example, Browning et al. 2013), and it is often difficult to make progress without it. Nevertheless, it is important to acknowledge that our data cannot rule out selection on preferences or the contingency of preferences. Moreover, recent empirical work by Brugler (2016) shows that consumption preferences systematically differ between never-married and divorced singles, which strongly suggests selection into marriage.

Despite these caveats, our conclusion is that, among potential explanations for the Deaton–Paxson puzzle, one should not rule out returns to scale in home production of food with heterogeneity in the time intensity of different foods.

## Electronic supplementary material

Appendix

## Footnotes

1 A number of explanations for this puzzle have been put forward, but none has proved satisfactory. Bulk discounts could mean that quantities consumed are higher in larger households, even though expenditures are lower (Abdulai 2003). However, Gibson and Kim (2016) show that careful estimates of bulk-discounts schedules based on transaction level data from Papua New Guinea are much too small to explain the Deaton–Paxson puzzle. In a developing country (the U.K.), Griffith et al. (2009) find evidence of larger bulk discounts, but report that demographic variables (including household size) explain less than 1% of the variation across households in savings from bulk purchasing. Gibson and Kim (2007) suggest that recall error in food expenditure data that is correlated with household size could explain the puzzle. That explanation has not been supported by subsequent analysis in Gibson et al. (2015) and Brzozowski et al. (2016). See Deaton (2010) for a more recent statement of the puzzle. For an excellent recent survey of the broader literature on home production of food, see Davis (2014).

2 It is worth noting that what we—following Deaton and Paxson—refer to as “returns to scale” is commodity scaling by demographics, following the Barten Model. Given a home production function for food at home, one could alternatively consider a more classical notion of the returns to scale of that production function as *inputs* are scaled. Davis and You (2013) report a recent attempt to estimate this more classical notion of the returns to scale in the production of food at home. They find increasing returns to scale in the time input.

3 Deaton and Paxson (1998) (and Gan and Vernon 2003) assume a more general form for the returns to scale ($\frac{x}{{\phi (n)}}$). Note however that we restrict our empirical analysis to singles and couples. With just two household sizes, a simple technology is completely general (even a linear technology would do).

4 Gorman (1976) famously wrote “When you have a wife and baby, a penny bun costs three pence.”

5 Deaton and Paxson (1998) note that this is particularly likely to be true in developing countries. They include such countries in their empirical analysis.

6 Deaton and Paxson find mixed results for alcohol and tobacco. These are goods for which it is quite plausible to assume that preferences change with household size (particularly co-habitation). Using data from the Canada Family Expenditure Survey 1992 and 1996, we find a positive but insignificant effect of household size (among singles and couples, and as always holding per capita income constant) for clothing, and a negative effect for alcohol and tobacco (significant only for the former).

7 Of course, this also means lower per capita time expenditures would not necessarily contradict the model, as this observation could be consistent with increasing effective time per person.

8 Prepared foods include cooked meats; canned meats; stews and soups; frozen precooked fish; canned pasta products; baked beans; pre-cooked meat or poultry pies; pre-cooked frozen dinners; other pre-cooked food preparations.

9 See Table 2 of Deaton and Paxson (1998).

10 In practice, this correction makes no difference to our results.

11 Among other things, these should account for any price effects.

12 These results are available in a working paper (Crossley and Lu 2005).

13 Deaton and Paxson (1998) condition on per capita expenditure, and then instrument this quantity with cash income, which is assumed to be exogenous.

14 In contrast, meals eaten in restaurants may comprise a bundle of different services, including entertainment.

15 Unit values are not quite a price, because variation in unit values can reflect, for example, variation in quality. See Deaton (1997) for further discussion.

16 Deaton and Paxson (1998) suggest the opposite, that unit values may rise with household size, citing evidence from Prais and Houthakker (1955).

17 Unfortunately, the GSS time use survey only reports household income in categories, precluding the possibility of conditioning on per capita income as we did in the food data.
