# Supplementary material for: Returns to scale in food preparation and the Deaton–Paxson puzzle
Source: Rev Econ Househ. 2017 Dec 2;16(1):5–19. doi: 10.1007/s11150-017-9399-4 (PMC6407849; doi:10.1007/s11150-017-9399-4)
Supplement: Supplementary file 1 — Appendix [file 11150_2017_9399_MOESM1_ESM.doc]

**Online Appendix: Full Regression Results**

**Table 1: Food Expenditure** Regressions

|  | (1) | | (2) | | (3) | | (4) | |
| --- | --- | --- | --- | --- | --- | --- | --- | --- |
|  | Food (purchased from store) budget share | | Ratio of prepared food to ingredients | | Ratio of take-out fast-food to ingredients | | Ingredients budget share | |
|  | coefficient | t-statistics | coefficient | t-statistics | coefficient | t-statistics | coefficient | t-statistics |
| couple | -0.011 | -3.80 | -0.103 | -4.01 | -0.110 | -3.21 | -0.007 | -2.96 |
| ln(per capita income) | -0.089 | -6.13 | -0.021 | -0.76 | 0.066 | 1.74 | -0.074 | -5.72 |
| age | 0.002 | 1.57 | -0.028 | -1.69 | -0.024 | -1.46 | 0.002 | 1.62 |
| age square | 0.000 | -0.96 | 0.000 | 1.38 | 0.000 | 1.02 | 0.000 | -0.95 |
| female | -0.005 | -1.51 | -0.021 | -0.82 | -0.070 | -2.27 | -0.003 | -1.19 |
| education level (base=high school or less) |  |  |  |  |  |  |  |  |
| some post-secondary | -0.001 | -0.33 | -0.064 | -1.75 | 0.091 | 1.83 | 0.001 | 0.37 |
| post-secondary certificate | 0.003 | 0.63 | -0.081 | -2.68 | 0.011 | 0.28 | 0.004 | 1.16 |
| university degree | 0.013 | 2.15 | -0.094 | -2.16 | -0.025 | -0.58 | 0.015 | 2.80 |
| Quarter (base=quarter 1) |  |  |  |  |  |  |  |  |
| 2 | 0.004 | 1.10 | -0.005 | -0.21 | -0.002 | -0.05 | 0.003 | 0.93 |
| 3 | 0.000 | -0.03 | 0.011 | 0.40 | 0.000 | -0.01 | 0.000 | -0.07 |
| 4 | -0.002 | -0.70 | 0.053 | 1.19 | 0.055 | 1.15 | -0.002 | -0.66 |
| region (base=Ontario) |  |  |  |  |  |  |  |  |
| Atlantic | -0.011 | -2.75 | 0.030 | 0.63 | -0.007 | -0.18 | -0.009 | -2.68 |
| Quebec | -0.002 | -0.56 | -0.078 | -2.59 | -0.005 | -0.08 | 0.000 | 0.09 |
| Prairies | -0.002 | -0.68 | -0.020 | -0.57 | -0.038 | -0.97 | -0.001 | -0.28 |
| British Columbia | -0.002 | -0.44 | -0.055 | -1.60 | -0.096 | -2.73 | 0.001 | 0.24 |
| year 1996 | 0.000 | -0.06 | -0.006 | -0.24 | -0.026 | -0.92 | -0.002 | -0.71 |
| constant | 0.938 | 6.06 | 1.386 | 2.91 | 0.261 | 0.59 | 0.771 | 5.54 |

Notes:

1. Based on a pooled sample of 1,201 singles and 956 childless couples from the 1992 and 1996 Canadian Food Expenditure Surveys. All members are aged 25-55 and working full time. In all calculations the data are weighted to equalize the proportion of each gender amongst singles.

Table 3: Meat unit values, expenditures and quantities

|  | (1) | | (2) | | (3) | | (4) | | (5) | |
| --- | --- | --- | --- | --- | --- | --- | --- | --- | --- | --- |
|  | Share of Prepared Meat in Total Meat ($) | | Share of Prepared Meat in Total Meat (£) | | Unit value total meat ($) | | Total Meat $ Per Capita | | Total Meat Kg per capita | |
|  | coefficient | t-statistics | coefficient | t-statistics | coefficient | t-statistics | coefficient | t-statistics | coefficient | t-statistics |
| couple | -0.063 | -4.08 | -0.072 | -4.65 | -0.207 | -1.56 | -1.294 | -2.69 | -0.178 | -1.71 |
| ln(per capita income) | 0.008 | 0.43 | 0.009 | 0.47 | 0.395 | 2.20 | 1.777 | 2.94 | 0.202 | 1.48 |
| age | 0.004 | 0.49 | 0.006 | 0.61 | 0.058 | 0.73 | 0.040 | 0.15 | -0.026 | -0.41 |
| age square | 0.000 | -0.81 | 0.000 | -0.89 | -0.001 | -0.98 | 0.001 | 0.29 | 0.001 | 0.75 |
| female | -0.038 | -2.47 | -0.040 | -2.58 | -0.139 | -1.02 | -0.178 | -0.40 | -0.060 | -0.62 |
| education level (base=high school or less) |  |  |  |  |  |  |  |  |  |  |
| some post-secondary | -0.037 | -1.62 | -0.032 | -1.41 | 0.329 | 1.55 | -0.628 | -0.85 | -0.214 | -1.25 |
| post-secondary certificate | -0.020 | -0.97 | -0.019 | -0.90 | 0.122 | 0.67 | -1.309 | -2.00 | -0.252 | -1.59 |
| university degree | -0.035 | -1.50 | -0.029 | -1.25 | 0.933 | 4.31 | -1.968 | -2.87 | -0.581 | -3.58 |
| Quarter (base=quarter 1) |  |  |  |  |  |  |  |  |  |  |
| 2 | -0.010 | -0.46 | -0.001 | -0.06 | -0.043 | -0.23 | 0.620 | 0.92 | 0.099 | 0.62 |
| 3 | 0.027 | 1.22 | 0.033 | 1.50 | 0.023 | 0.12 | 0.111 | 0.17 | -0.079 | -0.51 |
| 4 | -0.012 | -0.53 | -0.012 | -0.52 | 0.029 | 0.14 | 0.642 | 0.83 | 0.171 | 0.92 |
| region (base=Ontario) |  |  |  |  |  |  |  |  |  |  |
| Atlantic | -0.037 | -1.56 | -0.030 | -1.27 | -0.935 | -4.83 | 0.737 | 1.06 | 0.316 | 1.78 |
| Quebec | -0.044 | -1.89 | -0.028 | -1.21 | 0.148 | 0.75 | 0.598 | 1.09 | -0.087 | -0.78 |
| Prairies | 0.003 | 0.12 | 0.004 | 0.17 | 0.062 | 0.30 | 1.522 | 2.22 | 0.261 | 1.74 |
| British Columbia | -0.042 | -1.63 | -0.043 | -1.67 | 0.226 | 0.96 | 0.119 | 0.18 | -0.021 | -0.13 |
| year 1996 | 0.001 | 0.07 | 0.014 | 0.89 | -0.231 | -1.74 | 0.131 | 0.29 | 0.105 | 1.03 |
| constant | 0.315 | 1.28 | 0.247 | 1.00 | 2.072 | 0.99 | -11.243 | -1.44 | -0.209 | -0.12 |

Notes:

1. Based on a pooled sample of 959 singles and 884 childless couples with positive meat purchases from the 1992 and 1996 Canadian Food Expenditure Surveys. All members are aged 25-55 and working full time. In all calculations the data are weighted to equalize the proportion of each gender amongst singles.

Table 4: *Per Capita* Time use Regressions

|  | (1) | | (2) | | (3) | | (4) | |
| --- | --- | --- | --- | --- | --- | --- | --- | --- |
|  | Food Preparation + Clean up | | Food Preparation = (3) + (4) | | Meal Preparation | | Grocery Shopping | |
|  | coefficient | t-statistics | coefficient | t-statistics | coefficient | t-statistics | coefficient | t-statistics |
| couple | 9.54 | 2.78 | 6.70 | 2.19 | 8.84 | 3.34 | -2.14 | -1.49 |
| income category (base=$20,000-$49.999) |  |  |  |  |  |  |  |  |
| less than $20,000 | -3.27 | -0.74 | 0.73 | 0.18 | -2.53 | -0.75 | 3.26 | 1.42 |
| $50,000-$79,999 | -3.31 | -0.91 | -2.45 | -0.75 | -3.15 | -1.13 | 0.70 | 0.46 |
| $80,000 and more | 0.00 | 0.00 | 0.24 | 0.05 | -4.51 | -1.12 | 4.75 | 2.06 |
| age | 2.88 | 1.66 | 2.76 | 1.79 | 2.47 | 1.92 | 0.30 | 0.41 |
| age square | -0.03 | -1.44 | -0.03 | -1.60 | -0.03 | -1.72 | 0.00 | -0.35 |
| female | 18.37 | 5.83 | 13.41 | 4.82 | 11.08 | 4.83 | 2.33 | 1.84 |
| education level (base=less than 9 years) |  |  |  |  |  |  |  |  |
| some post-secondary | -2.66 | -0.56 | -2.71 | -0.65 | -1.45 | -0.42 | -1.26 | -0.68 |
| post-secondary certificate | 0.33 | 0.08 | -0.93 | -0.24 | 0.48 | 0.15 | -1.41 | -0.79 |
| university degree | -1.69 | -0.35 | -3.34 | -0.79 | -1.12 | -0.31 | -2.22 | -1.16 |
| Quarter (base=quarter 1) |  |  |  |  |  |  |  |  |
| 2 | -3.71 | -0.91 | -3.12 | -0.85 | -2.55 | -0.87 | -0.57 | -0.32 |
| 3 | -3.57 | -0.84 | -3.49 | -0.90 | -2.50 | -0.75 | -1.00 | -0.54 |
| 4 | -7.57 | -2.06 | -6.91 | -2.10 | -4.96 | -1.87 | -1.95 | -1.09 |
| region (base=Ontario) |  |  |  |  |  |  |  |  |
| Atlantic | -8.58 | -1.96 | -9.06 | -2.29 | -6.85 | -2.06 | -2.21 | -1.17 |
| Quebec | 6.91 | 1.37 | 1.27 | 0.29 | 0.63 | 0.19 | 0.64 | 0.30 |
| Prairies | 3.56 | 0.93 | 3.31 | 0.96 | 5.43 | 1.93 | -2.12 | -1.13 |
| British Columbia | 3.96 | 0.88 | 3.04 | 0.77 | 1.02 | 0.32 | 2.02 | 0.96 |
| constant | -26.89 | -0.82 | -24.58 | -0.84 | -27.68 | -1.14 | 3.10 | 0.22 |

Notes:

1. Based on a sample of 861 singles and 550 childless couples from the 1996 Canadian General Social Survey. All members are aged 25-55 and working full time. The data are weighted to equalize the proportion of each gender amongst singles.
